# Cardiovascular Risk Factors and Carotid Intima Media Thickness: Mediation and Interaction by Grip Strength

**DOI:** 10.1093/geroni/igab046.1481

**Published:** 2021-12-17

**Authors:** Christian Mendo, Mark Keezer, Marie-Pierre Sylvestre

**Affiliations:** 1 Universite de Montreal, Montreal, Quebec, Canada; 2 McGill University, Montreal, Quebec, Canada; 3 Université de Montréal, Montreal, Quebec, Canada

## Abstract

Frailty is often described as being an increased vulnerability to the effects of stressors. There is little research investing how frailty may act as either a mediator or participate in interactions in the associations between risk factors and chronic disease. We will present novel analyses of the Canadian Longitudinal Study on Aging, focusing on the 30,000 study participants who underwent serial physical evaluations at one of 11 data collection sites between 2011 and 2018. Using the 4- way decomposition method elaborated by Vanderweele, we investigate the role of grip strength, as a component of physical frailty, in the effect of cardiovascular risk factors on the atherosclerotic burden of individuals (measured using carotid intima media thickness). Our findings clarify the mechanisms underlying of grip strength in the associations between cardiovascular risk factors and carotid intima media thickness.

